# ‘Should a mammographic screening programme carry the warning: Screening can damage your health!’?

**DOI:** 10.1038/sj.bjc.6690111

**Published:** 1999-02

**Authors:** H Thornton, M Baum

**Affiliations:** ‘Saionara’ , 31 Regent Street, Rowhedge, Colchester, CO5 7EA, UK; Department of Surgery, Royal Free and University College Medical School, Charles Bell House, 67–73 Riding House Street, London, W1P 7LD, UK

**Keywords:** screening, citizens’, jury, democratic decision-making, information provision, psychological morbidity

## Abstract

The balanced presentation afforded by convening a Citizens' Jury when considering a major question such as the introduction of a breast screening programme is advocated. This method would enable account to be taken of all the costs, both human and financial, to all those affected, both participating and organizing, as well as the benefits. Provision of such a democratic opportunity enables consideration to be given to a broad range of factors, by selection of an appropriate range of witnesses, with the advantage of involving the lay public in this decision-making process. Attendance by health correspondents, medical journalists and other media representatives enables publicization of a democracy in action whilst helping to inform the wider debate. Such an exercise could inform whether the NHS BSP should continue in its current form. © 1999 Cancer Research Campaign


*British Journal of Cancer* (1999) **79**(5/6), 691–692
© 1999 Cancer Research Campaign
Article no. bjoc.1998.0111

Editorial

### ‘Should a mammographic screening programme carry the warning: Screening can damage your health!’?

**H Thornton[1] and M Baum[2]**

[1]‘Saionara’, 31 Regent Street, Rowhedge, Colchester CO5 7EA; [2]Royal Free and University College Medical School, Department of Surgery, Charles Bell House, 67–73 Riding House Street, London W1P 7LD, UK

**Summary** The balanced presentation afforded by convening a Citizens’ Jury when considering a major question such as the introduction of a breast screening programme is advocated. This method would enable account to be taken of all the costs, both human and financial, to all those affected, both participating and organizing, as well as the benefits.

Provision of such a democratic opportunity enables consideration to be given to a broad range of factors, by selection of an appropriate range of witnesses, with the advantage of involving the lay public in this decision-making process. Attendance by health correspondents, medical journalists and other media representatives enables publicization of a democracy in action whilst helping to inform the wider debate. Such an exercise could inform whether the NHS BSP should continue in its current form.

**Keywords:** screening; citizens’ jury, democratic decision-making; information provision; psychological morbidity

> *Screening has a distorted public belief. In our desire for good population coverage we have said that screening is simple, effective and inexpensive. In truth, it is complex, of limited effectiveness, and very expensive.*
>
> (Raffle, 1998)

The intention of this editorial is to contrast the appropriateness of organizing a Citizens’ Jury (Stewart et al, 1994) to help reach a decision on screening with the UK experience of the introduction of the National Health Service Breast Screening Programme (NHS BSP) a decade ago, drawing attention to the different prevailing attitudes at that time, contrasting the ‘Forrest’ autocratic era with the ‘Calman’ collaborative era acknowledging interdependence. It is our intention to suggest that the Citizens’ Jury method of providing a democratic opportunity to consider such a major question would, if well organized, not only involve the lay public in the decision-making process regarding an important population health care measure, but would be likely to provide, by careful and appropriate selection of witnesses, a balanced presentation of the many factors that ought to be considered before the initiation of such a programme.

We also suggest that with the benefits of hindsight on the NHS BSP UK experience a re-evaluation of the true benefits/cost ratio of the screening programme in the UK is in order, viewed not solely from the point of view of the benefits in terms of a possible mortality reduction – i.e. to those individuals that *benefit* from screening – but by taking account of *all the costs*, both financial and human, to all those affected by organization of, and participation in, the programme (Thornton, 1998). It is with wistful regret that we acknowledge that such an example was not available for our Ministers of Health and health professionals in the climate of the 1980s. Whilst acknowledging that the NHS BSP was in the forefront of providing a well-intentioned and quality service in attempts to deal with the scourge of breast cancer, we should now be seeking out all the evidence (Thornton, 1997) to ask ourselves what are the true successes and failures of our attempt. More importantly, should we be asking if the NHS BSP should continue in its current form?

We would agree with a recent editorial in the *New England Journal of Medicine* (Sox, 1998) commenting on a recent 10-year retrospective cohort study (Elmore et al, 1998) of breast cancer screening and diagnostic evaluation among 2400 American women who were between 40 and 69 years old at study entry which said that ‘most people like to make important decisions about health care on the basis of current facts rather than myth or peer pressure’. This study found a near 50% cumulative risk of a false positive result after 10 mammograms. These false positive results led to 870 outpatient appointments, 539 diagnostic mammograms, 186 ultrasound examinations, 188 biopsies and 1 hospitalization. We concur with the idea that ‘those who counsel women about screening mammographs should learn the facts and convey them accurately’. The Panel Report of the National Institutes of Health Consensus Development Conference contained the crucial sentences: ‘Each woman should decide for herself whether to undergo mammography’ (Sox, 1998) and that ‘Physicians should educate women about the risk of a false positive result of a screening test for breast cancer’ (Elmore, 1998).

In order to emphasize the importance of as wide as possible a consideration of evidence, suggestions for categories of witness are offered as follows: someone to provide the rationale/purpose of screening; a member of the expert advisory group to the Ministers; an epidemiologist; the Director of our NHS BSP; someone with practical experience of running a model screening centre; a clinician/trialist with experience of directing a wide screening service; an intelligent, thoughtful radiologist.

*Received 20 March 1998*
*Revised 3 July 1998*
*Accepted 17 August 1998*

*Correspondence to:* H Thornton

This editorial is based on a paper presented at the First Presidential Symposium of the British Oncological Association, 2 March 1998 at the Royal Society of Medicine

Other suggestions are: a breast cancer patient who believes her life was saved – perhaps a member of an advocacy group; a breast cancer patient with asymptomatic screen-detected ductal carcinoma in situ (DCIS); a breast cancer surgeon; a health economist; a Health Minister, or perhaps the ex-Health Minister of 1989; a manufacturer of radiography equipment; a sociologist or social scientist interested in Public Health or an Academic in Health Services Management. This suggestion is prompted by Sarah Stewart-Brown's piece in the *British Medical Journal* (Stewart-Brown and Farmer, 1997) entitled 'Screening could seriously damage your health. Decisions to screen must take account of the social and psychological costs.'

Also required would be an ethicist versed in the World Health Organization's Principles of Screening, as well as a mathematician. Michael Retsky of the University of Colorado, Colorado Springs, USA is suggested because he is engaged in mathematical modelling of breast cancer. His model predicts that only 15% of patients with T1 tumours would benefit from adjuvant chemotherapy, compared with 51% of those with T3 tumours. Retsky says: 'Ironically this means that adjuvant chemotherapy and early detection may not be compatible strategies. This has implications for screening. As smaller and smaller tumours are found, with better long-term prognosis, therapies become less and less effective, reducing the overall gains of early detection' (Bonn, 1997).

It is recommended that a spokesperson for the 1% of the population that is heterozygous for the ataxia telangiectasia gene (*ATM*), where affected women have a fivefold increased risk of breast cancer, should give evidence. They have an induced increase in radiosensitivity which has implications for procedures such as chest and dental radiography, mammography and radiation treatment. Ursula Werneke (1997) has pointed out that the risk–benefit ratio may be acceptable for therapeutic procedures targeted at individual patients, but is not acceptable for prevention programmes for sections of whole population, as in, for example, mammographic screening.

To enlarge on the suggestion that a radiographer or a member of a breast screening team be called is to give the opportunity to present evidence on a rather unexpected aspect of the statement that 'Screening can damage your health'. In this case it is the health of the professionals within the breast screening team. Such a witness would enlarge upon the pitfalls and problems associated with the withdrawal of ring-fenced funding. Enormous stresses can result from cut-backs in departments managed by those who do not understand the need for proper staffing and resources for its organization, including the provision of proper information and the risk of being named in the House of Commons if your false negative rate is transiently raised.

Information providers have to tread an impossibly fine line as the 'window-dressers' in the 'shop-window' of screening programmes, which are a reversal of the usual doctor/patient relationship. They aim for a target of 70% uptake to make the programme work, thus dividing their loyalty between the programme and the patient. When this fine line crumbles, it not only damages the health of the numerous participants who were the inevitable false negatives and false positives, but the doctors' own health as well. They may have seen the quality of their department eroded by inadequacies, and the threats or realities of litigation looming. As the *Lancet* recently said in its editorial (Lancet, 1998) concerning cervical screening where the comments might equally well apply to the breast screening programme, 'The UK information leaflets and letters … are a model of research based wording and design, yet only allude to the concept of false test result, with little or no explicit explanation of what a false result *means*.' They may ponder that those who are damaged by the programme did not appreciate the significance of such banal phrases as 'the test is not 100% perfect' or 'is not 100% accurate', which are deemed, after much re-drafting, to be sufficient indication of the inevitable false test results to a population used to coercive healthism, who believe screening is good for you and a responsible course of action. But, as Angela Raffle said of cervical screening, 'Screening has a distorted public belief.'

Those participants who have suffered dearly and may pay the ultimate price having experienced a false negative will feel they have been duped and their intelligence insulted as they re-read the leaflets that only alluded to the concept of false test results without explicit explanation of what a false result actually means. Likewise, so may the recipient of a false positive result who may have suffered invasive tests and treatment when neither is needed. In their cases, the psychological morbidity is considerable as these healthy participants – and their families – wait for results of tests. As Calman appreciates, carers too can be damaged by sub-optimal cancer care – even diagnostic! He also appreciates that patients, families and carers must be given clear information and that communication between sectors must be of high quality if the best possible care is to be achieved (Calman and Hine, 1994).

The triple combination of a less than perfect test and a less than perfect provision caused by less than perfect information can cause triple damage: to screened patients, relations/friends/carers, and the screening team.

Health correspondents and medical journalists and other representatives from the media may be invited to attend Citizens' Juries with the advantage that this publicizes democracy in action and informs debate.

We cannot prejudge the outcome of the new trial in front of a fresh jury, but we would prefer to stand by that verdict rather than the one of 10 years ago when the evidence was less complete.

### REFERENCES

Bonn D (1997) Bringing numbers to bear in breast-cancer therapy. *Lancet* **350**: 104
Calman K, Hine D (1994) A Policy Framework for Commissioning Cancer Services, May 1994. Prepared by an Expert Advisory Group on Cancer to the Chief Medical Officers of England and Wales
Elmore JG, Barton MB, Moceri VM, Polk S, Arena PJ, Fletcher SW (1998) Ten-year risk of false positive screening mammograms and clinical breast examinations. *N Engl J Med* **338**: 1089–1096
Editorial: The screening muddle. *Lancet* (1998) **351**: 459
Raffle A (1998) Letter: New test in cervical screening. *Lancet* **351**: 297
Sox HC (1998) Benefit and harm associated with screening for breast cancer. *N Engl J Med* **338**: 1145–1146
Stewart-Brown S, Farmer A (1997) Screening could seriously damage your health. Decisions to screen must take account of the social and psychological costs. *Br Med J* **314**: 533
Stewart J, Kendall E, Coote A (1994) Citizens' Juries. Institute for Public Policy Research (IPPR), London
Thornton H (1997) The voice of the breast cancer patient – a lonely cry in the wilderness. *Eur J Cancer* **33**: 825–828
Thornton H (1998) Letter: The cervical screening muddle. *Lancet* **351**: 1129
Werneke U (1997) Ataxia telangiectasia and risk of breast cancer. *Lancet* **350**: 739–740

*© Cancer Research Campaign 1999*

